# The Effects of Selective Dorsal Rhizotomy on Balance and Symmetry of Gait in Children with Cerebral Palsy

**DOI:** 10.1371/journal.pone.0152930

**Published:** 2016-04-04

**Authors:** Franziska Rumberg, Mustafa Sinan Bakir, William R. Taylor, Hannes Haberl, Akosua Sarpong, Ilya Sharankou, Susanne Lebek, Julia F. Funk

**Affiliations:** 1 Paediatric Orthopaedic Surgery and Neuroorthopaedics, Department of Orthopaedics, Center for Musculoskeletal Surgery (CMSC), Charité –Universitätsmedizin Berlin, Berlin, Germany; 2 Institute for Biomechanics, ETH Zürich, Switzerland; 3 Section of Paediatric Neurosurgery, Department of Neurosurgery, University of Ulm, Ulm, Germany; 4 Social Paediatric Center, Charité –Universitätsmedizin Berlin, Berlin, Germany; 5 Julius Wolff Institute, Charité –Universitätsmedizin Berlin, Berlin, Germany; IRCCS E. Medea, ITALY

## Abstract

**Aim:**

Cerebral palsy (CP) is associated with dysfunction of the upper motor neuron and results in balance problems and asymmetry during locomotion. Selective dorsal rhizotomy (SDR) is a surgical procedure that results in reduced afferent neuromotor signals from the lower extremities with the aim of improving gait. Its influence on balance and symmetry has not been assessed. The aim of this prospective cohort study was to evaluate the impact of SDR on balance and symmetry during walking.

**Methods:**

18 children (10 girls, 8 boys; age 6 years (y) 3 months (m), SD 1y 8m) with bilateral spastic CP and Gross Motor Function Classification System levels I to II underwent gait analysis before and 6 to 12 months after SDR. Results were compared to 11 typically developing children (TDC; 6 girls, 5 boys; age 6y 6m, SD 1y 11m). To analyse balance, sway velocity, radial displacement and frequency were calculated. Symmetry ratios were calculated for balance measures and spatio-temporal parameters during walking.

**Results:**

Most spatio-temporal parameters of gait, as well as all parameters of balance, improved significantly after SDR. Preoperative values of symmetry did not vary considerably between CP and TDC group and significant postoperative improvement did not occur.

**Interpretation:**

The reduction of afferent signalling through SDR improves gait by reducing balance problems rather than enhancing movement symmetry.

## Introduction

Muscle tone abnormalities in children with cerebral palsy (CP) lead to deficits in selective motor control resulting in difficulties with coordination, gait efficiency and symmetry, as well as posture and balance [1,2]. About 90% of muscle tone abnormalities in children with CP are spastically induced [3]. Spasticity, together with muscular imbalance and reduced neuromuscular control during targeted task performance, leads to weakness despite high muscle tone. The complex motor impairments in CP children are thought to originate from the hyper-excitability of reflexes–related to excessive afferent signalling–that results in a consecutive loss of inhibitory impulses [4]. The human brain normally counterbalances excitatory signals from the sensory nerves with inhibitory electric signals [5]. In cases of cerebral or spinal damage, this balance mechanism is perturbed and the excessive sensory signals are thought to lead to spasticity [6]. Spasticity and weakness, as positive and negative features of the upper motor neuron syndrome respectively [5], are both thought to affect postural control and thus both balance and symmetry in subjects with CP [7]. Due to an impaired neuromuscular response, together with delayed onset of contraction in CP, an increased co-contraction is observed during balance, resulting in slower and less organized muscular coordination [8]. In order to assess the extent of this condition, impaired balance in children with CP has been evaluated during standing [7–11] but also more recently during gait [12–14].

The maturation of balance skills in children with cerebral palsy is also known to be delayed or diminished when compared to typically developing children [15]. These deficits have been demonstrated through the longer time to recover from a stability disturbance and an increased centre of pressure (CoP) movement after perturbation [8,16]. Asymmetry of gait patterns may also lead to further functional impairment in ambulatory CP children [17,18] since increasingly symmetric gait improves walking efficiency, provides easier dynamic balance control and decreases unilateral strain on the joints of the lower limbs [19].

With the goal of permanently diminishing spasticity and improving motor function of the lower limbs, a selective dorsal rhizotomy (SDR) may be performed in selected patients, whereby lumbosacral sensory nerve rootlets are partially severed to reduce their exaggerated excitatory afferent signalling. Although the reduction of spasticity and improvement of motor function after SDR have been described by several authors, the relevance and longevity of the positive results are still discussed controversially [20–29]. Data concerning changes in gait patterns and function are less available after SDR, although the subjective improvement is known to be immense [29–33]. As a consequence, the aim of this study was to evaluate the changes that occur to balance and gait symmetry after SDR using accurate and objective methods.

## Methods

### Patients

Eighteen children with bilateral spastic cerebral palsy and a Gross Motor Function Classification System (GMFCS) level I and II who underwent selective dorsal rhizotomy (L1 to S2) as a standard of care procedure for reducing spasticity using a single-level laminoplasty approach as described by Funk and Haberl [34] (a modification of Park’s method [35]) were included in this single-arm study (Table 1). The percentage of rootlets cut was between 50 and 60 per cent for each level and each side. The decision which rootlets to cut was taken based on evaluation of the electromyography (EMG) signals according to Philipps and Park [36]. Functional movements and kinematic patterns were not taken into account when cutting the rootlets. All of the children had multilevel spasticity of the lower limbs prior to surgery and only patients who were able to complete an instrumented 3D gait analysis preoperatively were recruited into this study (S1 Fig).

**Table 1:** CP children’s and TDC characteristics.

	TDC (n = 11)		CP pre SDR (n = 18)		CP post SDR (n = 18)	
	Value	SD	Value	SD	Value	SD
**Age at SDR**	6y 6m	1y 11m	6y 3m	1y 8m	7y 1m	1y 6m
**Gender (male/female)**	5/6		8/10		8/10	
**Height (cm)**	122.6	13.1	114.7	11.4	123.3	6.3
**Weight (kg)**	22.6	5.7	20.4	6.4	22.0	3.0
**Leg length (cm)**	62.6	10.1	56.5	5.7	61.1	4.7
**GMFM**	not tested		89.2	6.0	91.1	7.9
**MAS**^a^	0	0	1.4	0.3	0.7	0.4
**Strength**^b^	5.0	0	3.2	0.6	3.6	0.6

TDC = typically developing children, CP = cerebral palsy, SDR = selective dorsal rhizotomy, SD = standard deviation, y = year, m = months, GMFM = Gross Motor Function Measure, MAS = Modified Ashworth Scale.

^a^ Averaged measurements of spasticity of hip adductors, knee flexors, and ankle plantar flexors.

^b^ Averaged measurements on a 5 point scale of hip extension, abduction, knee extension, ankle dorsiflexion and plantarflexion.

To be considered for SDR, strict selection criteria were applied [37]. Children with impairment of the extrapyramidal system or skeletal deformities were excluded. The final study cohort included subjects with a mixture of bilateral spastic walking types according to the classification of Rodda and co-workers [38]. Between March 2010 and May 2012 the children were recruited for this study. Kinematic and kinetic data was recorded during barefoot walking, collected between 6 and 12 months postoperatively so that the time of follow-up lasted until May 2013. The mean age of the CP children at the day of surgery was 6 years 3 months with a standard deviation (SD) of 1 year 8 months. The treatment included a minimum of 3 week’s rehabilitation with the aim to retrain walking so that at least short distances could be covered after discharge from the rehabilitation centre [28]. After SDR, all eighteen children returned for post-op follow-up gait analysis, with an average follow-up of 8.6 (6–12) months postoperatively. Between SDR and the follow-up gait analysis no further intervention was performed other than each child’s individual therapy programme, including orthotic treatment. In addition, a cohort of 11 asymptomatic typically developing children (TDC) with a mean age of 6 years 6 months (SD 1 year 11 months) was recruited to act as a control. Informed written consent was obtained from the children’s parents for all procedures, and the study was approved by the ethics committee of the Charité - Universitätsmedizin Berlin (EA1/138/11, S1 File). The study was not registered primarily in a trial registry as it is part of an ongoing clinical evaluation which was not required to be registered according local guidelines. The authors confirm that it was retrospectively registered at DRKS—German Clinical Trials Register—for all ongoing and related trials for this intervention (DRKS00004798; S2 File). All measurements were post-processed non-blinded.

### Balance

Ground reaction forces were recorded using two triaxial force plates at 960Hz (AMTI OR6-7-1000, Watertown, MA, USA). Trials were recorded with both feet approximately parallel and at shoulder width apart, standing with both feet on one force plate and afterwards with one foot on each force plate. The children were instructed to stand as still as possible with their arms in a relaxed standing position, without making macro-movements with their feet or upper body, and while focussing on a cross on the wall at a distance of approximately 3 m. Data was filtered for high-frequency noise with a low-pass fifth-order Butterworth digital filter with a cut off frequency of 10 Hz according to Rose and Wolf [9,39].

Total sway path velocity (mm/s), its bidirectional components anterior-posterior (A-P) and medio-lateral (M-L) sway (mm/s) as well as the mean radial sway frequency (MRF; Hz) and the average radial displacement (ARD; mm) were calculated using the CoP according to Rose and co-workers [9]. Sway path velocity can be considered the rate of movement of the CoP across the force plate, and hence be representative of the velocity of the sway of the body centre of mass (CoM) while standing still. ARD describes the average displacement of the CoP from the centre of balance of the trial, hence describing the average distance the body moves from its calculated middle. The MRF provides a measure of the rate at which the CoP revolves around the centroid with the defined radial displacement at the given sway path velocity.

### Symmetry

Instrumented gait analysis was performed using a 10 camera three-dimensional motion capture system (Vicon, Oxford Metrics Group, Oxford, UK) at 120 Hz. A retro-reflective marker set (marker diameter 14mm) was placed on the participant’s skin by an experienced observer following a standardized protocol [40]. Gait analysis was performed while the subjects walked barefoot along a 10 meter straight and level walkway at a self-selected speed. At least five undisturbed trials with 5 strides each were recorded and analysed for each child at each session [32]. The data were processed using Vicon Nexus 1.7. (Vicon, Oxford Metrics Group, Oxford, UK). Gait events, including initial contact and toe-off were detected via an adapted version of the foot velocity algorithm [41], which used the heel markers of each foot to enable a reliable definition of the gait cycle, even in cases of toe-first contact [42]. Stride, step, stance and swing phases, as well as double limb support were computed on the basis of those two events [43]. Foot progression angle (FPA) was measured as the angle of the longitudinal foot axis to the movement direction at mid-stance. Walking base was determined as the sum of the M-L distances of the right and left feet from the line of progression, where the line of progression was defined by the direction of the anterior-posterior axis of motion over each complete gait cycle. Cadence was defined as the number of strides per second. Stride height described the maximum of the vertical component of the heel. The spatial parameters were normalised for leg length according to the method of Hof to account for growth of the children between the measurements [44], and are thus presented as dimensionless parameters. On the basis of the absolute spatio-temporal parameters and the previously described balance measures (X), the symmetry ratios (SR) were calculated as: SR = X_higher value_/ X_lower value_ as described by Patterson [19].

### Statistical analysis

As the aim of this study was to analyse the effect of SDR on motor function, only the CP children’s results were evaluated statistically. The results of the TDC group were presented descriptively as means and standard deviations for comparison. An a priori power analysis was performed on the basis of the data of Rose et al. [9]. At least 14 participants would be required to reach an alpha of 0.05 and a power of 0.80. Statistical data analysis was performed using SPSS version 20 (v20, IBM, Champaign, IL, USA). The level of significance was set at p<0.05. All parameters were tested for normal distribution with the Kolmogorov-Smirnov-test. Normally distributed variables were then compared using the Student-t-test for paired variables.

## Results

Preoperatively, all evaluated absolute parameters of CP children’s gait, except cadence, differed considerably from those observed in TDC (Table 2). After SDR most absolute spatiotemporal parameters improved significantly (p = 0.004–0.047) and aligned partially with the parameters of the TDC group.

**Table 2:** Absolute spatiotemporal values and radial balance parameters.

	TDC			CP pre-SDR			CP post-SDR			p-value
	Mean	SD	95% CI	Mean	SD	95% CI	Mean	SD	95% CI	
**Duration of Gait cycle (N)**	3.55	0.41	3.28–3.83	4.15	1.37	3.46–4.83	3.79	0.66	3.46–4.12	0.177
**Duration of Stance Phase (%)**	54.6	5.3	51.0–58.1	64.6	6.7	61.3–67.9	61.9	3.1	60.4–63.5	**0.031**
**Duration of Swing Phase (%)**	34.6	3.2	32.5–36.8	35.4	6.7	32.1–38.7	38.1	3.1	38.1–39.6	**0.031**
**Duration of Double limb support (%)**	10.0	1.5	8.9–10.9	14.6	6.5	11.4–17.9	11.9	3.2	10.4–13.5	**0.025**
**Stride length (N)**	1.52	0.17	1.40–1.63	1.14	0.26	1.01–1.27	1.33	0.21	1.23–1.44	**0.009**
**Stride height (N)**	0.32	0.02	0.30–0.33	0.30	0.02	0.29–0.30	0.31	0.02	0.30–0.32	**0.005**
**Step length (N)**	0.78	0.07	0.73–0.83	0.69	0.12	0.63–0.75	0.74	0.10	0.70–0.79	**0.047**
**Walking Base (N)**	0.15	0.07	0.10–0.19	0.36	0.09	0.31–0.40	0.30	0.09	0.25–0.34	**0.004**
**FPA at mid-stance (deg)**	16.8	5.5	13.1–20.5	-8.7	18.0	(-17.9)-0.6	4.7	16.2	(-3.4)-12.8	**0.010**
**Velocity (N)**	0.45	0.09	0.39–0.50	0.32	0.11	0.26–0.37	0.37	0.09	0.33–0.42	**0.027**
**Cadence (N)**	0.59	0.07	0.53–0.62	0.53	0.12	0.47–0.59	0.55	0.08	0.51–0.59	0.563
**ARD BL (mm)**	7.0	1.6	5.9–8.0	12.0	3.5	10.1–13.9	8.0	3.1	6.3–9.8	**0.001**
**ARD UL-R (mm)**	7.3	2.5	5.6–8.9	10.3	3.0	8.6–12.0	6.9	2.4	5.5–8.3	**0.002**
**ARD UL -L (mm)**	6.0	2.7	4.2–7.8	9.6	4.4	7.2–12.1	6.2	2.8	4.7–7.8	**0.000**
**MRF BL (Hz)**	0.55	0.12	0.47–0.63	0.37	0.11	0.31–0.43	0.50	0.11	0.44–0.57	**0.000**
**MRF UL-R (Hz)**	0.55	0.14	0.46–0.64	0.39	0.13	0.33–0.46	0.52	0.15	0.44–0.59	**0.006**
**MRF UL-L (Hz)**	0.61	0.14	0.52–0.70	0.41	0.16	0.33–0.49	0.59	0.18	0.50–0.68	**0.000**

Paired samples t-test for normally distributed data; TDC = typically developing children, CP = cerebral palsy, SDR = selective dorsal rhizotomy, SD = standard deviation, CI = confidence interval, N = normalised for leg length, % = percentage of stride time or percentage of related gait cycle phase, FPA = foot progression angle, deg = degree (negative values describe in-toeing, positive values out-toeing), ARD = average radial displacement, MRF = mean radial frequency of sway, BL = bilateral, UL = unilateral, R = right, L = left.

### Balance

**Fig 1:**
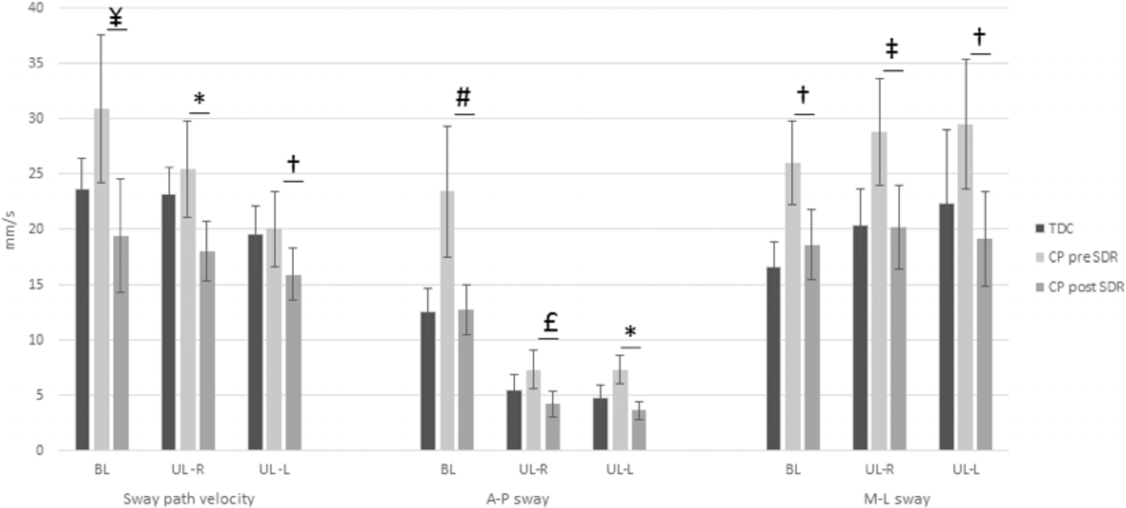
Changes of velocity related balance parameters. Paired samples t-test for normally distributed data; TDC = typically developing children, CP = cerebral palsy, SDR = selective dorsal rhizotomy, BL = bilateral, UL = unilateral, R = right, L = left, * p = 0.000, † p = 0.001, ‡ p = 0.002, # p = 0.004, £ p = 0.006, ¥ p = 0.007.

Similar to the changes observed in gait patterns, SDR led to a significant improvement in all evaluated balance parameters (Table 2 and Fig 1). Both sway path velocity (p = 0.000–0.007) and bidirectional (A-P and M-L) sway (p = 0.000–0.006) decreased significantly after SDR in all measurements while it was observed that all children including TDC swayed more in the M-L direction than in the A-P direction (Fig 1). In addition, the MRF of sway increased significantly (p = 0.000–0.006) and the ARD decreased significantly (p = 0.00–0.002), also approaching the values of the typically developing children (Table 2).

### Symmetry

The results of the symmetry evaluation showed that most spatiotemporal parameters were close to perfect symmetry (SR = 1), even in the preoperative state. As a result, no significant changes were found between conditions (Table 3). Although the asymmetry of the postural sway parameters (sway path velocity, A-P and M-L sway, ARD and MRF) and the foot progression angles (FPA) were found to be more pronounced in all evaluated groups, a significant improvement after SDR was not detected.

**Table 3:** Symmetry ratio of spatiotemporal and postural balance parameters.

	TDC			CP pre-SDR			CP post-SDR			p-value
	Mean	SD	95% CI	Mean	SD	95% CI	Mean	SD	95% CI	
**Gait cycle**	1.01	0.01	1.00–1.01	1.01	0.01	1.01–1.01	1.01	0.01	1.01–1.01	0.696
**Stance Phase**	1.02	0.01	1.01–1.03	1.03	0.04	1.01–1.05	1.04	0.03	1.03–1.05	0.525
**Swing Phase**	1.02	0.02	1.01–1.03	1.07	0.07	1.03–1.11	1.06	0.04	1.04–1.08	0.678
**Double limb support**	1.11	0.08	1.06–1.16	1.16	0.18	1.07–1.25	1.20	0.33	1.03–1.36	0.702
**Stride length**	1.01	0.01	1.00–1.01	1.01	0.01	1.00–1.01	1.01	0.01	1.00–1.01	1.000
**Stride height**	1.03	0.03	1.01–1.05	1.07	0.05	1.04–1.09	1.06	0.06	1.03–1.09	0.585
**Step length**	1.03	0.02	1.01–1.04	1.08	0.07	1.04–1.11	1.06	0.06	1.03–1.10	0.503
**Walking Base**	1.06	0.08	1.01–1.12	1.02	0.05	1.00–1.05	1.03	0.02	1.02–1.04	0.761
**FPA at mid-stance**	2.38	1.51	1.36–3.39	4.51	7.31	0.75–8.26	11.27	29.59	-3.45–25.98	0.358
**FPA at initial contact**	1.20	0.35	0.96–1.44	3.33	3.99	1.35–5.31	2.27	2.10	1.22–3.31	0.265
**Sway path velocity**	1.14	0.09	1.08–1.20	1.30	0.32	1.15–1.45	1.33	0.32	1.18–1.48	0.548
**Average radial displacement (ARD)**	1.62	0.62	1.21–2.04	1.63	0.77	1.27–2.00	1.50	0.47	1.28–1.72	0.527
**A-P sway**	1.28	0.26	1.10–1.45	1.89	1.08	1.38–2.39	1.87	0.69	1.55–2.20	0.665
**M-L sway**	1.63	0.50	1.29–1.96	1.60	0.78	1.24–1.96	1.54	0.51	1.30–1.78	0.804
**Mean radial frequency (MRF)**	1.61	0.45	1.31–1.91	1.37	0.62	1.08–1.66	1.19	0.16	1.11–1.26	0.213

Paired samples t-test for normally distributed data; TDC = typically developing children, CP = cerebral palsy, SDR = selective dorsal rhizotomy, SD = standard deviation, CI = confidence interval, FPA = foot progression angle, A-P sway = Anterior-posterior sway, M-L sway = medio-lateral sway.

## Discussion

Cerebral Palsy is the most common disorder inducing motor impairment in early childhood, and often results in the delayed onset of gait and impaired motor control [1,2]. Locomotion including standing without support is a task that requires a high level of coordination between spinal reflexes, positioning and movement control. Typical CP gait shows a co-activation and synchronisation of muscle groups rather than a gait cycle adapted temporally coordinated activation of agonist and antagonist muscles of the lower limb [45]. In addition, CP children show a lack of selective control mechanisms, velocity dependent muscle recruitment (spasticity), and various muscle-tendon-unit mal-alignments [7,45]. These motor constraints lead to decreased gait velocity, cadence, and step length, asymmetrical gait patterns and impaired postural stability [7]. Hence, quantification and comparison of these parameters provides a logical and critical basis for evaluating the efficacy of interventions that are performed with the intention to improve locomotion patterns.

Numerous studies have described the effects of SDR with regard to spasticity, gait and function [21,22,24,25,33,37,46–53]. Although our results are in general agreement with the spatio-temporal parameters described in these studies, this is the first investigation exploring the impact of SDR on balance and symmetry of gait in children with CP. Interestingly, key metrics of gait and balance were improved through SDR, but symmetry was not, possibly due to the highly symmetrical preoperative gait patterns of the children. SDR intervenes with controlling mechanisms on the spinal level and is therefore likely to have an impact on the basic, alternating activation of flexors and extensors generated at the spinal level. In addition, exact task control, fine motor skills and postural control, which are all influenced by both afferent signalling and cerebral entities, are almost certain to be affected. Due to the known mechanisms of monosynaptic reflexes and corticospinal tract control [6], as well as the variable distribution of the efferent innervation, a general lumbosacral reduction of spasticity seems to be more likely to change the motor control and functioning than a sectioning of the afferences with regard to clinical tests of spasticity or typical patterns in gait analysis. Our study is the first to demonstrate that the reduction of afferent input through SDR reduces balance impairments in children with CP GMFCS levels I and II. As this effect was observed as soon as the children were mobile again after surgery, the relatively short follow-up of our study group clearly demonstrates the efficacy of the therapy at an early time point. However, the longevity of the effects has to be confirmed in investigations with a long-term follow-up. As the children in this study did not have significant contractures or lever-arm disease it was assumed that the changes in balance and movement patterns observed were induced by the surgically altered neurological pathways and not by mechanical alteration.

In contrast to TDC, where postural control normally improves with age, CP children do not show this functional improvement [39] without treatment [9]. With improved balance, as indicated by significantly lower A-P and M-L sway values in the SDR cohort, it is possible that CP children might develop higher motor skills during walking [14,54] and recover more easily from unexpected perturbations [7]. Thus, as a major finding of this study, SDR, which appears to enhance balance skills, might be a surgical option to enable CP children to participate in more demanding activities. Whether this improvement is able to affect children with different levels of motor function or different gait patterns in the same way could not be shown in this study due to the small number of patients, but there is certainly cause for optimism due to the improvement observed for every child in this study group. Energy efficiency while moving is a common underlying principle in nature that requires a high degree of inter-limb symmetry during gait. Its existence is often overlooked or even taken for granted in many clinical and research settings [55]. Improved balance is thought to contribute to more efficient movement, as it is known that a reduction in the lateral and vertical deviation of the centre of mass (CoM) acts to reduce energy costs [56].

The energy cost of a healthy individual’s gait is known to be reduced through a reduction of sway of the CoM and associated symmetrical gait patterns [57]. These postural control mechanisms are impaired in children with CP and might constitute a major component of gait disorders in CP [58,59]. Additionally, CP children show an increased background muscle activity during static stance, take longer to recover stability, and move their CoM significantly more when recovering balance [8,60]. Thomas and co-workers [33] found higher levels of subjective endurance after SDR, but did not associate this with improved balance, which might be a significant contributor to energy efficient locomotion. Here, the improvement of cost efficiency and endurance might not be visible in spatio-temporal gait parameters in the confined surroundings of a gait laboratory.

The lack of significant improvement in gait symmetry through SDR in our study cohort is thought to at least partly result from the patient selection procedure, as surgery was indicated by our interdisciplinary team only in children with bilateral spastic CP, without musculoskeletal deformities, with good postural trunk control, and rather consistent preoperative gait patterns [35,37]. Therefore, our patient cohort walked reasonably symmetrically, even at the preoperative time point. Although symmetry of movement as one prerequisite for efficient motor task performance has been evaluated in disorders such as stroke or CP [55,57,61–65], information on the impact of surgical interventions on gait symmetry in CP children does not exist to date. However, the question to what extent symmetry is centrally controlled, but also what impact contractures and mechanical impairments have on gait symmetry and postural control, cannot be answered within this study.

If interventions are expected to influence gait performance, parameters describing intrinsic factors of cost efficiency such as balance, symmetry, and variability [32] are likely to provide valuable additional information on the achieved alterations of the underlying central processing. For the selection of optimal treatment options in ambulatory CP children, these parameters might help to differentiate between spasticity, weakness, and poor motor control [53].

## Conclusions

The extent of the impact of balance and symmetry control on locomotion in ambulatory patients with motor disorders has not yet been thoroughly studied. However, the clear and significant impact of SDR on balance and gait in CP shown in our study demonstrates the critical importance that a full understanding and characterisation of these parameters should be gained. The findings should be taken into account when planning interventions for ambulatory children with motor impairments. Especially for patients with a high functional level, as in this study, changes in functional outcome might be more thoroughly described—but further investigation in less functional cohorts is clearly still required. Our study provides strong evidence of a positive impact of SDR on motor skills and gait performance in children with CP and therefore an improvement of their quality of movement. The efficacy of this procedure for long term benefits, however, remains to be demonstrated.

## Supporting Information

S1 Fig CONSORT 2010 Flow Diagram for the Recruitment of Children. (PDF)

S1 File Study protocol. (DOCX)

S2 File TREND Statement Checklist. (PDF)
