# Correction: The Effects of Selective Dorsal Rhizotomy on Balance and Symmetry of Gait in Children with Cerebral Palsy

**DOI:** 10.1371/journal.pone.0177585

**Published:** 2017-05-08

**Authors:** Franziska Rumberg, Mustafa Sinan Bakir, William R. Taylor, Hannes Haberl, Akosua Sarpong, Ilya Sharankou, Susanne Lebek, Julia F. Funk

## Notice of republication

This article was republished on April 11, 2017, as the original S1 File was published in error. Please download this article again to view the correct version.
