# Supplementary material for: The Effects of Selective Dorsal Rhizotomy on Balance and Symmetry of Gait in Children with Cerebral Palsy
Source: PLoS One. 2016 Apr 4;11(4):e0152930. doi: 10.1371/journal.pone.0152930 (PMC4820221; doi:10.1371/journal.pone.0152930)

CONSORT 2010 Flow Diagram for the Recruitment of Children for the Evaluation of the Effect of Selective Dorsal Rhizotomy on Balance and Symmetry of Gait

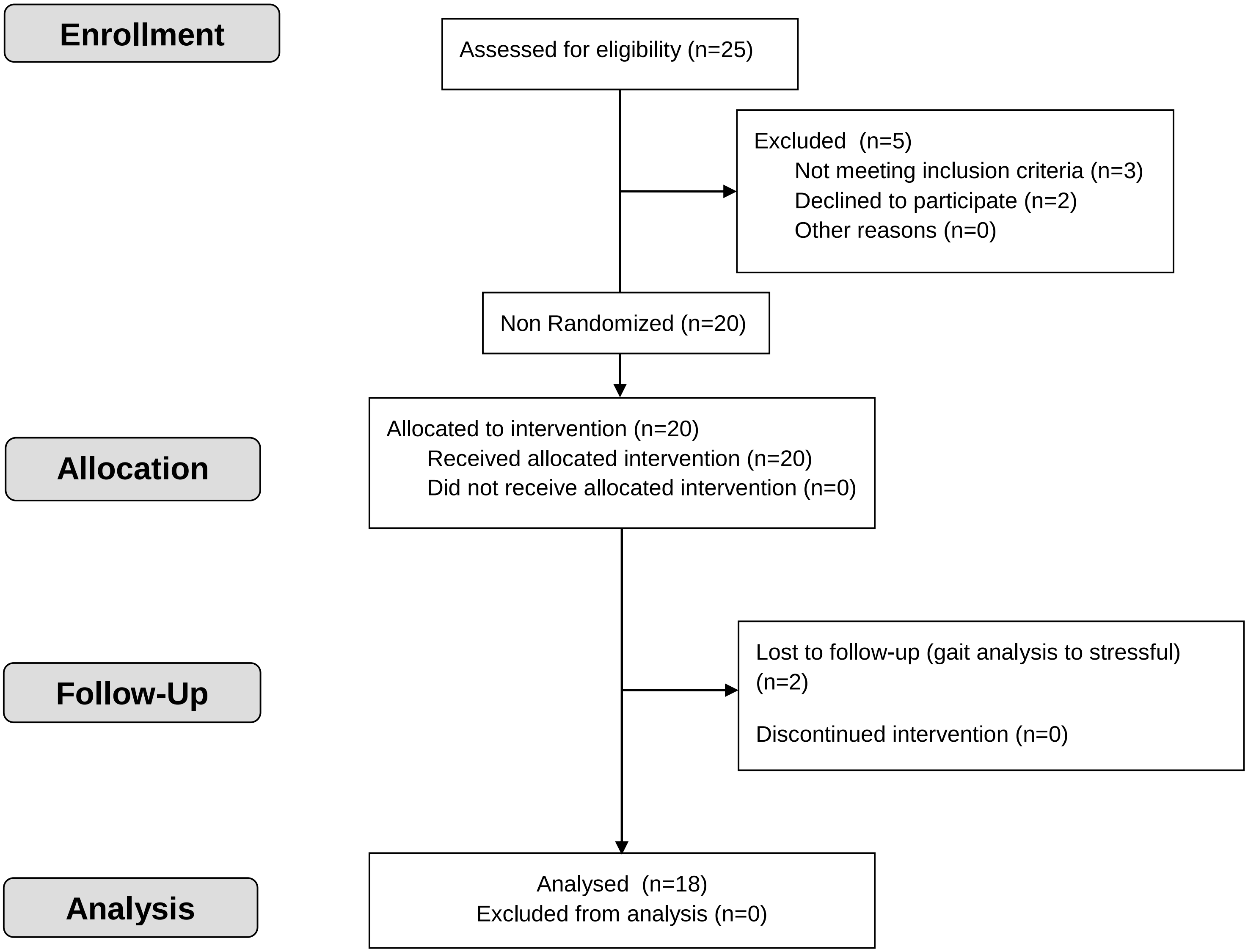

Supplement: S1 Fig — (PDF) [file pone.0152930.s001.pdf]
