# Supplementary material for: The Effects of Selective Dorsal Rhizotomy on Balance and Symmetry of Gait in Children with Cerebral Palsy
Source: PLoS One. 2016 Apr 4;11(4):e0152930. doi: 10.1371/journal.pone.0152930 (PMC4820221; doi:10.1371/journal.pone.0152930)
Supplement: S1 File — (DOCX) [file pone.0152930.s002.docx]

| \| Charité ⏐ Campus Virchow-Klinikum ⏐ 13353 Berlin \| \| --- \|   **Centrum für Muskuloskeletale Chirurgie**  **Klinik für Orthopädie**  **Klinik für Unfall- und Wiederherstellungschirurgie**  Direktor: Univ.-Prof. Dr. med. Dr. h. c. Norbert P. Haas  **Sektion Kinder- und Neuroorthopädie**  stellv. Leitung: Dr. med. J. Funk  Campus Virchow-Klinikum  Augustenburger Platz 1  13353 Berlin |
| --- | --- |

**Charité Centrum für Unfall- und Wiederherstellungschirurgie**

Berlin, 09.03.2010

**Untersuchungsprotokoll zur neuroorthopädisch-neuropädiatrischen Verlaufskontrolle nach selektiver dorsaler Rhizotomie**

***Examination protocol for neuroorthopedic-neuropediatric follow-up after selective dorsal rhizotomy***

**SDR Study Protocol**

**Conduct plan**

|  | **0 Months** | **3 Months** | **6 Months** | **12 Months** | **24 Months** | **60 Months** |
| --- | --- | --- | --- | --- | --- | --- |
| GMFM | x |  |  | x | x | x |
| MAS | x | x | x | x | x | x |
| MFT | x | x | x | x | x | x |
| ROM | x | x | x | x | x | x |
| GA | x | x |  | x | x | x |
| XRAY | x |  |  | x | x | x |
| HRQOL | x |  |  | x | x | x |

**Checklist SDR**

| **Name**: | DOB: | SDR day of surgery: |
| --- | --- | --- |
|  |  |  |

| **Preoperative Examinations** | | | | | | | |
| --- | --- | --- | --- | --- | --- | --- | --- |
| GMFM | | Ashworth | | | | | |
| total | Target dim. | Adductors | | hamstrings | | M. triceps surae | |
|  |  | right | left | right | left | right | left |
|  |  |  |  |  |  |  |  |
| Gait analysis (ventral /dorsal /sagittal)  Please mark  □ barefoot  □ with insoles/orthotics  □ completed | | | | | | | |
| Examiner: | | | | | | | |

| **3 Months Examinations** | | | | | | | |
| --- | --- | --- | --- | --- | --- | --- | --- |
| GMFM | | Ashworth | | | | | |
| total | Target dim. | Adductors | | hamstrings | | M. triceps surae | |
|  |  | right | left | right | left | right | left |
|  |  |  |  |  |  |  |  |
| Gait analysis (ventral /dorsal /sagittal)  Please mark  □ barefoot  □ with insoles/orthotics  □ completed | | | | | | | |
| Examiner: | | | | | | | |

| **6 Months Examinations** | | | | | | | |
| --- | --- | --- | --- | --- | --- | --- | --- |
| GMFM | | Ashworth | | | | | |
| total | Target dim. | Adductors | | hamstrings | | M. triceps surae | |
|  |  | right | left | right | left | right | left |
|  |  |  |  |  |  |  |  |
| Gait analysis (ventral /dorsal /sagittal)  Please mark  □ barefoot  □ with insoles/orthotics  □ completed | | | | | | | |
| Examiner: | | | | | | | |

| **12 Months Examinations** | | | | | | | |
| --- | --- | --- | --- | --- | --- | --- | --- |
| GMFM | | Ashworth | | | | | |
| total | Target dim. | Adductors | | hamstrings | | M. triceps surae | |
|  |  | right | left | right | left | right | left |
|  |  |  |  |  |  |  |  |
| Gait analysis (ventral /dorsal /sagittal)  Please mark  □ barefoot  □ with insoles/orthotics  □ completed | | | | | | | |
| Examiner: | | | | | | | |

| **24 Months Examinations** | | | | | | | |
| --- | --- | --- | --- | --- | --- | --- | --- |
| GMFM | | Ashworth | | | | | |
| total | Target dim. | Adductors | | hamstrings | | M. triceps surae | |
|  |  | right | left | right | left | right | left |
|  |  |  |  |  |  |  |  |
| Gait analysis (ventral /dorsal /sagittal)  Please mark  □ barefoot  □ with insoles/orthotics  □ completed | | | | | | | |
| Examiner: | | | | | | | |

| **60 Months Examinations** | | | | | | | |
| --- | --- | --- | --- | --- | --- | --- | --- |
| GMFM | | Ashworth | | | | | |
| total | Target dim. | Adductors | | hamstrings | | M. triceps surae | |
|  |  | right | left | right | left | right | left |
|  |  |  |  |  |  |  |  |
| Gait analysis (ventral /dorsal /sagittal)  Please mark  □ barefoot  □ with insoles/orthotics  □ completed | | | | | | | |
| Examiner: | | | | | | | |

| **Additional Examination date:** | | | | | | | |
| --- | --- | --- | --- | --- | --- | --- | --- |
| GMFM | | Ashworth | | | | | |
| total | Target dim. | Adductors | | hamstrings | | M. triceps surae | |
|  |  | right | left | right | left | right | left |
|  |  |  |  |  |  |  |  |
| Gait analysis (ventral /dorsal /sagittal)  Please mark  □ barefoot  □ with insoles/orthotics  □ completed | | | | | | | |
| Examiner: | | | | | | | |

Determination of muscle tone with the Ashworth scale

This examination is carried out before the joint angle measurement. The examination should always be performed by the same examiner. The patient lies in supine position, the head slightly elevated on a pillow (10cm). The measurement of the adductors occurs in hip and knee extension. The anterior superior spines are marked. The pelvis is fixed with one hand, while the other leg is abducted slowly. This movement is performed during one second, and repeated 3 times. The measurement of the hamstrings muscles occurs in hip flexion (90 °).

1. No increase in muscle tone
2. Slight increase in muscle tone, expressed as muscle tension and relaxation, or by minimal resistance at the end of range of motion, when affected body part / the affected body parts is / are flexed or extended
3. Slight increase in muscle tone, manifested in muscle tension, followed by minimal resistance throughout the remaining (less than half) range of motion
   1. More pronounced increase in muscle tone over the major part of the movement. But the affected parts can be moved easily
   2. Considerable increase in muscle tone, passive movement difficult
   3. The affected body part / the affected body parts are rigidly flexed or extended.

| Date: |  | Please insert scale code |
| --- | --- | --- |
| Adductors |  | \| Right \|  \| Left \| \| --- \| --- \| --- \| \|  \|  \|  \| \|  \|  \|  \| |
| Hamstrings |  | \|  \|  \|  \| \| --- \| --- \| --- \| \|  \|  \|  \| |
| M. triceps surae |  | \|  \|  \|  \| \| --- \| --- \| --- \| \|  \|  \|  \| |

# Muscle testing form (according to Daniels/Worthingham, Skale 0-5)

Name:

| Date |  | |  | |  | |  | |
| --- | --- | --- | --- | --- | --- | --- | --- | --- |
| **Lower Limb:** | right | left | right | left | right | left | right | left |
| Dorsal extension |  |  |  |  |  |  |  |  |
| Plantar flexion |  |  |  |  |  |  |  |  |
| Knee extension |  |  |  |  |  |  |  |  |
| Knee flexion |  |  |  |  |  |  |  |  |
| Hip extension |  |  |  |  |  |  |  |  |
| Hip flexion |  |  |  |  |  |  |  |  |
| Hip abduction |  |  |  |  |  |  |  |  |
| Hip adduction |  |  |  |  |  |  |  |  |

**Selective Control Assessment of the Lower Extremity (SCALE)**

1) Each joint is first moved by the examiner the limb **passively** within the desired directions of movement to demonstrate the task. The examiner talks during this movement for a duration of 3 seconds (for example, "Bend Stretch Bend". The passive range of motion will be recorded in order to compare it with the active range of motion can.

2) The patient is prompted to **actively** make the requested movement in approximately the same time (3 seconds) without moving other joints of the examined or the contralateral limb. If the patient should not succeed, the patient receives feedback. Further trials are allowed.

The patient is asked to ...

- In the hip joint: ... to bend the leg, to stretch the leg, to bend, (knee kept straight).

Examiner holds the leg, without supporting the motion.

(In patients with a pronounced tightening of the hamstrings it can be tested as an alternative if the hip can be stretched when the knee is held in flexion.)

- In the knee joint: ... to straighten the knee and to bend.

- in the upper ankle joint: ... to move the foot in dorsiflexion and plantar flexion.

(with extended knee joint)

- In the lower ankle joint: ... to move the foot in inversion and eversion.

- Toes: ... to move in flexion and extension

-

. **Test date**: __________________

| **Patient**: | | Passive (Neutral-Null-Method) | | Aktive | |
| --- | --- | --- | --- | --- | --- |
| Joint | Position | right | left | right | left |
| Hip | Lateral |  |  |  |  |
| Knee | Sitting |  |  |  |  |
| UAJ | Sitting |  |  |  |  |
| LAJ | Sitting |  |  |  |  |
| Toes | Sitting |  |  |  |  |
| Total points | |  |  |  |  |

**„Normal“(2)**: desired motion sequence is executed within the time frame without associated movements of other (not to be tested) joints of the lower limb (ipsilateral and contralateral)

**„** **Impaired“(1)**: isolated movement is partially visible; some of the following errors can be observed:

- Movement is in one direction only

- Observed motion corresponds to less than 50% of the achievable, passive range of motion

- Movement in not to be tested joint is observed (including mirror movements)

- Time for execution of the motion exceeds the 3-second count time

**„Not possible“ (0)**: required movement sequence is not initiated or executed by means of synergistic mass movements in flexion or extension;

Synergistic mass movements are defined as concurrent, obligate flexor or extensor patterns in two or more joints. If the patient does not initiate the desired movement, extension or flexion synergies can be triggered by manual resistance to check the power generation of the muscle

By adding the scores measured for the individual joints (maximum 10 points per limb) a SCALE-value is obtained for each limb.

| Name |  |  | Range of Motion Measurement | | ***Neutral-Null-Method*** | |  |  |  |
| --- | --- | --- | --- | --- | --- | --- | --- | --- | --- |
|  |  | Date: | | Date: | | Date: | | Date: | |
|  |  | Right | Left | Right | Left | Right | Left | Right | Left |
| Hip |  |  |  |  |  |  |  |  |  |
| Psoas-Shift |  |  |  |  |  |  |  |  |  |
| Lasègue |  |  |  |  |  |  |  |  |  |
| Flex – Ext  130° / 0 / 10° |  |  |  |  |  |  |  |  |  |
| IRO / ARO  Hip ext / Knee flex)  45° / 0 / 45° |  |  |  |  |  |  |  |  |  |
| ABD / ADD  (+ Knee flex)  45° / 0 / 30° |  |  |  |  |  |  |  |  |  |
| ABD / ADD  (+ Knee ext)  45° / 0 / 30° |  |  |  |  |  |  |  |  |  |
| Knee |  |  |  |  |  |  |  |  |  |
| Flex – Ext  (sup)  135 – 155° / 0 / 5° |  |  |  |  |  |  |  |  |  |
| Flex – Ext  (Hip flex 90°)  Popliteal angle | slow |  |  |  |  |  |  |  |  |
|  | quick |  |  |  |  |  |  |  |  |
| Ankle |  |  |  |  |  |  |  |  |  |
| DE – PF  20° / 0 / 45° | in Knee extension: slow |  |  |  |  |  |  |  |  |
|  | quick |  |  |  |  |  |  |  |  |
|  | in Knee flexion: slow |  |  |  |  |  |  |  |  |
|  | quick |  |  |  |  |  |  |  |  |

| Video Gait Analysis Name: | | | | | | | | | |
| --- | --- | --- | --- | --- | --- | --- | --- | --- | --- |
|  | Date |  | |  | |  | |  | |
| Gait parameter |  | right | left | right | left | right | left | right | left |
| Knee position stance leg | flexion > 10°  flexion < 10°  neutral 0°  recurvatum > 5°  recurvatum < 5° | 0  1  2  0  1 | 0  1  2  0  1 | 0  1  2  0  1 | 0  1  2  0  1 | 0  1  2  0  1 | 0  1  2  0  1 | 0  1  2  0  1 | 0  1  2  0  1 |
| Gait | Fixed toe walking  Dynamic toe walking  Occasional heel contact  Plantigrade  Rockers of the heel | 0  1  2  3  4 | 0  1  2  3  4 | 0  1  2  3  4 | 0  1  2  3  4 | 0  1  2  3  4 | 0  1  2  3  4 | 0  1  2  3  4 | 0  1  2  3  4 |
| Base of gait | scissoring  narrow base (< hip distance)  wide base (> hip distance)  normal base | 0  1  2  3 | 0  1  2  3 | 0  1  2  3 | 0  1  2  3 | 0  1  2  3 | 0  1  2  3 | 0  1  2  3 | 0  1  2  3 |
| Speed | No tempo variation  variable (fast - slow) | 0  1 | 0  1 | 0  1 | 0  1 | 0  1 | 0  1 | 0  1 | 0  1 |
| Supporting device | Walker with additional help  Walker without help  Sticks/crutches/Tetrapodes  Unaided at least 10m | 0  1  2  3 | 0  1  2  3 | 0  1  2  3 | 0  1  2  3 | 0  1  2  3 | 0  1  2  3 | 0  1  2  3 | 0  1  2  3 |
| Total score |  |  |  |  |  |  |  |  |  |
| Total score |  |  | |  | |  | |  | |

**Attachment 2: List of Variables for Extended SDR-Evaluation**

For extended evaluation of pre-, intra- and postoperative data to assess the success of treatment following categories are statistically evaluated:

| **Variables** | **Scales** |
| --- | --- |
| Number | 3-digit |
| Code | 8-digit |
| Intracerebral diagnosis |  |
| Pre-term birth |  |
| gender | nominal (m/f) |
| age | metric (years) |
| weight | metric (kg) |
| height | metric (cm) |
| Duration of anesthesia | (min) |
| Duration of surgery | (min) |
| Complications intra-, peri-, postoperative |  |
| Surgeries prior to SDR |  |
| Surgeries after SDR |  |
| **Pre-/postoperative scores** |  |
| Dexterity / Laterality / Limb dominance | R-L |
| (Modified) Ashworth Skale | 0-4 |
| Tardieu-Skale | degree |
| Gross motor function measure (GMFM) | % |
| Gross Motor Function Classification System (GMFCS) | I-V |
| Ferrari-classification | I-IV |
|  |  |
| Passive range of motion Hip/Knee/Ankle Neutral-Null-Method in 3 dimensions (ROM) | degree |
| Popliteal angle | degree |
| Manual muscle force testing | Ordinal 0-5 |
| Selectivity | Ordinal 0-2 |
| Stance stability |  |
| Gait velocity | [cm/sec] |
| Cadence | [steps/min] |
| Step/stride length | m |
| Trunk movement | degree, N, Nm |
| Kinetics of pelvis/hip/knee/ankle in 3 dimensions during gait | Nm |
| Kinematics of pelvis/hip/knee/ankle in 3 dimensions during gait | degree |
| Internal forces on pelvis/hip/knee/ankle in 3 dimensions during gait | N |
| Foot progression angle | degree |
| Elektromyographic Data from EMGs | V, s, Hz |
| Reimers migration percentage radiographical | percent |
| Femoral anteversion clinical and radiographical | degree |
| Gillette Gait Index (GGI) |  |
| Gait Deviation Index (GDI) |  |
| Gait Profile Score (GPS) |  |
| Movement Analysis Profile (MAP) |  |
| Tibial torsion clinical | degree |
| Cobb angle radiographical (Skoliosis) | degree |
| Consolidation of the Laminoplasty | nominal |
| Intraoperative Data from SDR-EMG-Protocol (per segment and total) |  |
| Amount of tested rootlets (absolute/relative/median;right/left) |  |
| Amount of severed rootlets (absolute/relative/median;right/left) |  |
| Amount of „Grade 1-4“ tested rootlets (absolute/relative/median;right/left) |  |
| Amount of severed „Grad1-4“ tested rootlets (absolute/relative/median;right/left)) |  |
| Amount of rootlets with Grade 0 (absolute/relative/median;right/left) |  |
| Amount of rootlets with Grade 1 (absolute/relative/median;right/left) |  |
| Amount of rootlets with Grade 1+2 (absolute/relative/median;right/left) |  |
| Amount of rootlets with Grade 2 (absolute/relative/median;right/left) |  |
| Amount of rootlets with Grade 3 (absolute/relative/median;right/left)) |  |
| Amount of rootlets with Grade 3+4 (absolute/relative/median;right/left) |  |
| Amount of rootlets with Grade 4 (absolute/relative/median;right/left) |  |
| Amount of roots ONLY with Grade 0 (absolute/relative/median;right/left) |  |
| Amount of roots totally without Grade 0 (absolute/relative/median;right/left) |  |
| Maximum measured Grade (right/left) |  |
| Segmental Distribution of „Grade 1-4“ answers (right/left) |  |
| Evaluation of Innervation of Muscle groups per Root (right/left) |  |
| Amount of Roots with low threshold intensity (right/left) |  |
| Amount of Areflexia after Stimulation with maximum 10mA (right/left) |  |
| Amount of contralateral answers following root-Stimulation (right/left) |  |
| Amount of asymmetric Distribution of pDRAP (S1, S2, S3) (right/left) |  |
|  |  |
|  |  |
